# *Corynebacterium propinquum*: A Rare Cause of Prosthetic Valve Endocarditis

**DOI:** 10.1155/2016/1391789

**Published:** 2016-11-07

**Authors:** Umair Jangda, Ankit Upadhyay, Farshad Bagheri, Nilesh R. Patel, Robert I. Mendelson

**Affiliations:** Jamaica Hospital Medical Center, Department of Medicine, Jamaica, NY 11418, USA

## Abstract

Nondiphtheria* Corynebacterium* species are often dismissed as culture contaminants, but they have recently become increasingly recognized as pathologic organisms. We present the case of a 48-year-old male patient on chronic prednisone therapy for rheumatoid arthritis with a history of mitral valve replacement with prosthetic valve. He presented with fever, dizziness, dyspnea on exertion, intermittent chest pain, and palpitations. Transesophageal echocardiography revealed two medium-sized densities along the inner aspect of the sewing ring and one larger density along the atrial surface of the sewing ring consistent with vegetation. Two separate blood cultures grew* Corynebacterium propinquum*, which were sensitive to ceftriaxone but highly resistant to vancomycin and daptomycin. The patient completed a course of ceftriaxone and repeat TEE study and after 6 weeks demonstrated near complete resolution of the vegetation. To our knowledge, this case represents the first in the literature of* Corynebacterium propinquum* causing prosthetic valve endocarditis. The ability of these organisms to cause deep-seated systemic infections should be recognized, especially in immune-compromised patients.

## 1. Introduction

Prosthetic valve endocarditis (PVE) remains a rare but very serious complication of valve replacement. The incidence of prosthetic valve endocarditis ranges from 1% to 6% of valve implantations [1]. The most commonly reported pathogens causing PVE are coagulase-negative staphylococci,* Staphylococcus aureus*, and Gram-negative bacilli [2].* Corynebacterium propinquum* is primarily isolated from the human respiratory tract [3]. It has been shown to be the pathogenic organism in two cases of native valve infective endocarditis, one in an adult and one in a child with congenital heart disease [4, 5]. It was also shown to be the causative agent in a handful of respiratory tract infections worldwide [6–8]. We present the case of a 48-year-old male with late PVE caused by* Corynebacterium propinquum*. To our knowledge, this case represents the first in the literature of* Corynebacterium propinquum* causing prosthetic valve endocarditis.

## 2. Case Report

A 48-year-old male presented with five to six weeks of dizziness with episodes of subjective fever. Patient also reported having increasing dyspnea on exertion associated with intermittent sharp chest pain, unrelated to exertion, and palpitations lasting 20–30 seconds per episode. Additionally, he complained of paroxysmal nocturnal dyspnea but denied orthopnea, peripheral edema, recent dental work, surgical procedure, or intravenous drug use. He has a significant past medical history of hypertension, rheumatoid arthritis on chronic low dose prednisone therapy, and rheumatic heart disease not on antibiotic prophylaxis. The patient had a mitral valve replacement with a bioprosthetic valve 27 years priorly, which was subsequently replaced with a metallic valve 12 years ago. The patient had a pacemaker placed for symptomatic bradycardia 12 years ago.

**Figure 1:**
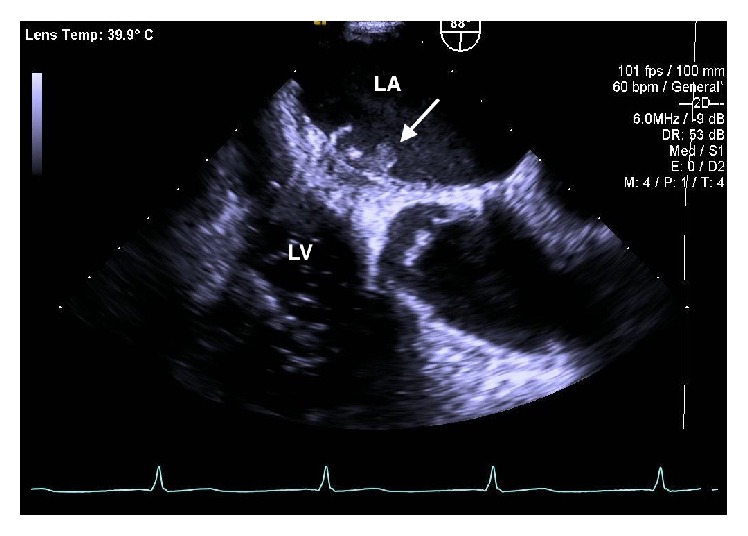
Transesophageal echocardiogram showing prosthetic mitral valve with vegetation (arrow). LA: left atrium; LV: left ventricle.

On admission, the patient was an ill appearing male and was complaining of a dull aching chest pain 4/10 in intensity. Vital signs were significant for fever of 101.6°F (38.6°C). Physical examination revealed tactile fever, clear breath sounds, and metallic S1. Laboratory findings showed a hemoglobin of 8.3 g/dL, leukocytosis of 17,900/uL with 91% PMNs, INR of 6.1, Troponin-I of 0.534 ng/mL, ESR of 85, and C-reactive protein of 20.70 mg/dL. Patient also had an elevated creatinine of 1.8 mg/dL. Prosthetic valve endocarditis was suspected and the patient received broad-spectrum antibiotics including vancomycin, cefepime, and gentamicin. Vancomycin was changed to daptomycin due to worsening renal function. Transesophageal echocardiography revealed mild left ventricular dysfunction with an ejection fraction of 50%. It also showed a mechanical prosthesis in the mitral position with two medium-sized, strand-like, echogenic, highly mobile densities along the inner aspect of the sewing ring and one larger echogenic, spherical density, measuring 9 × 5 mm along the atrial surface of the sewing ring, consistent with vegetation as shown in Figure 1. Two separate blood cultures drawn four hours apart from different sites on the initial day of presentation grew* Corynebacterium propinquum*.  Table 1 shows antimicrobial susceptibility of* Corynebacterium propinquum*.

**Figure 2:**
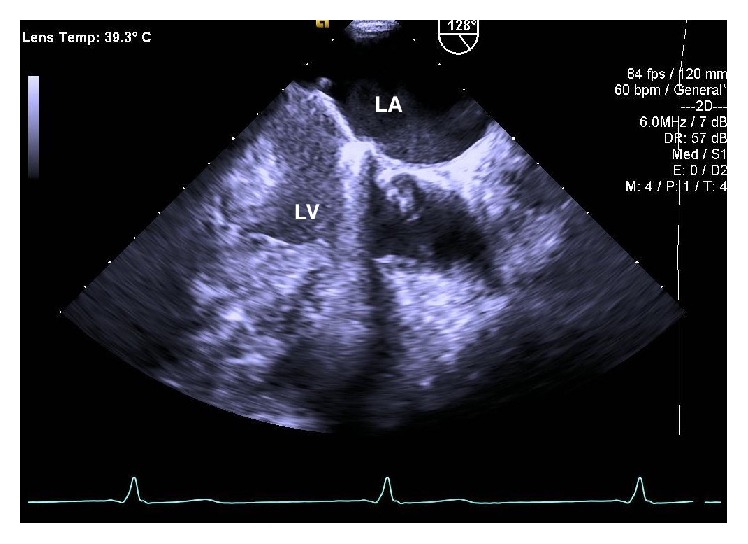
Repeat transesophageal echocardiogram repeated after six weeks of antibiotic treatment showing resolution of the vegetation. LA: left atrium; LV: left ventricle.

At the recommendation of the infectious disease and cardiology services, the patient was discharged home with a peripherally inserted central catheter to complete two months of intravenous ceftriaxone. Follow-up transesophageal echocardiogram done six weeks later demonstrated near complete resolution of the vegetation shown in Figure 2. Repeat blood cultures drawn at six weeks after starting antibiotic treatment were negative for any growth.

## 3. Discussion

The genus* Corynebacterium* has more than 80 published species of which over 50 can cause occasional or rare infections in humans [9].* Corynebacterium* species are normal colonizers of the skin and mucous membranes and are often dismissed as culture contaminants. It is thought that the incidence of nondiphtheria* Corynebacterium* is increasing, especially as a cause of nosocomial infections and infections in immunocompromised patients [3]. In our case, the patient was on prednisone, which could have contributed to development of infection with* Corynebacterium*. In recent literature, there have been an increasing number of reports of* Corynebacterium* species causing prosthetic valve endocarditis [10].

*Corynebacterium propinquum* is primarily isolated from the human respiratory tract [3]. It has been shown to be the pathogenic organism in two cases of native valve infective endocarditis, one in an adult and one in a child with congenital heart disease [4, 5]. It has also been reported as the causative agent in a handful of respiratory tract infections worldwide [6–8]. It has also been reported to cause acute nongonococcal urethritis in one patient from Iran [11]. To our knowledge, this is the first report of* Corynebacterium propinquum* causing prosthetic valve endocarditis. The susceptibility pattern for the strain of* C. propinquum* from our case showed high resistance to vancomycin and daptomycin, which is unusual for this species. As in other studies, it was susceptible to beta-lactams and an aminoglycoside as well as other antibiotics [4, 5]. Due to the rarity of infections caused by this bacteria, it would be premature to make assumptions regarding resistance patterns.

## 4. Conclusion

*Corynebacterium* species are becoming recognized as an increasing cause of opportunistic infections. In our case, the patient had risk factors of immune suppression as well as a prosthetic valve resulting in this very rare infection.* C. propinquum* has now been shown to be a cause of not only infective endocarditis but also prosthetic valve endocarditis and nosocomial infections. Physicians should be cognizant of potential serious infections caused by* C. propinquum* in the correct clinical context. Proper identification of* Corynebacterium* species and recognition of its ability to be pathologic is important in order to inform prompt and appropriate treatment.

## Figures and Tables

**Table 1:** Minimum inhibitory concentration for *Corynebacterium propinquum*.

Antimicrobial agent	MIC (*μ*g/mL)
Ceftriaxone	0.094
Ciprofloxacin	0.016
Daptomycin	>256
Imipenem	0.019
Tetracycline	0.125
Vancomycin	12
